# A Randomised, Double-Blind, Placebo-Controlled Crossover Trial of Resveratrol Supplementation for Prophylaxis of Hormonal Migraine

**DOI:** 10.3390/nu14091763

**Published:** 2022-04-22

**Authors:** Jemima S. A. Dzator, Peter R. C. Howe, Kirsten G. Coupland, Rachel H. X. Wong

**Affiliations:** 1 School of Biomedical Sciences and Pharmacy, University of Newcastle, Callaghan 2308, Australia; selorm.dzator@uon.edu.au (J.S.A.D.); kirsten.coupland@newcastle.edu.au (K.G.C.); rachel.wong@usq.edu.au (R.H.X.W.); 2 Adelaide Medical School, University of Adelaide, Adelaide 5005, Australia; 3 Centre for Health Research, University of Southern Queensland, Raceview 4305, Australia; 4 Hunter Medical Research Institute, New Lambton Heights 2305, Australia

**Keywords:** resveratrol, phytoestrogen, nutrient intervention, cerebrovascular function, hormonal migraine, prophylaxis, migraine disability assessment

## Abstract

Resveratrol, a vasoactive phytoestrogen, has beneficial effects on cerebrovascular function. Previous research has shown that hormonal migraineurs have poorer cerebrovascular function than non-migraineur women. We aimed to investigate if resveratrol supplementation for three months could reduce the hormonal migraine burden index (HMBI: the number of days with menstrual migraine per month), reduce migraine-related disability and improve migraine-related quality of life. A randomised, double-blind, placebo-controlled, crossover, intervention trial was conducted in 62 hormonal migraineurs (mean age: 37.5 ± 0.8 years). Participants consumed 75 mg of resveratrol or matching placebo capsules twice daily for three months before crossing over to the other treatment arm. Participants completed a daily diary and the Headache Impact Test-6™, Migraine Disability Assessment and Migraine-Specific Quality of Life questionnaires at months 0, 3 and 6. The HMBI was the primary outcome and was calculated using data extracted from the participant’s diary. No differences in the HMBI (*p* = 0.895), the Headache Impact Test-6™, the Migraine Disability Assessment and Migraine-Specific Quality of Life were found between the resveratrol and placebo treatments. Resveratrol supplementation for three months did not affect the HMBI, the migraine-related disability or quality of life measures in our cohort of hormonal migraineurs.

## 1. Introduction

Migraine is a neurovascular disorder so severe that it has been ranked as the seventh most disabling disease worldwide [1,2]. Characterised by a severe, throbbing, often unilateral headache that can last four to seventy-two hours, migraine affects one in ten people globally [3]. Approximately 1 in 2 women with migraine experience migraine attacks that occur when oestrogen concentrations rapidly decline in the days prior to menstruation [4,5]. These migraines are classified as hormonal migraines [5,6].

In a previous cross-sectional study conducted by our research group, we found that women with hormonal migraine had altered cerebrovascular function, represented by lower resting cerebral blood flow velocity and lower neurovascular coupling capacity during cognitive stimulation, when compared with women who suffered from no type of migraine at all. We also reported that the hormonal migraineurs in our cross-sectional study experienced severe migraine-related disability and that migraine was adversely affecting their quality of life [7]. We, therefore, hypothesised that by improving the cerebrovascular function of hormonal migraineurs, we may potentially prevent the hormonal migraine from occurring and ultimately reduce migraine-related disability and improve quality of life.

Resveratrol is a phytoestrogen found in grapes and berries that is known to have multiple targeted benefits including cardiovascular, anti-inflammatory, antioxidant and neuroprotective effects [8]. Previous research has shown that acute and chronic resveratrol can improve cerebrovascular function [9,10]. Kennedy et al. reported that 250 mg and 500 mg of acute resveratrol supplementation in 22 healthy adult participants was able to increase neurovascular coupling capacity during a cognitive task in a dose-dependent manner compared with placebo [10]. Additionally, in a 14-week randomised, double-blind, placebo-controlled, parallel study conducted by our research group, we evaluated the effects of resveratrol supplementation (75 mg capsule twice daily) in a population of postmenopausal women. Findings from this study showed that resveratrol supplementation was able to significantly improve neurovascular coupling capacity during a cognitive task battery [9]. There are several pathways by which resveratrol might exert this therapeutic benefit. Given that it is structurally and functionally similar to the active form of oestrogen, 17-β-estradiol, it may influence cerebrovascular function by acting on oestrogen receptors ERα and ERβ to increase cerebral perfusion by increasing production and bioavailability of nitric oxide. Resveratrol may also modulate vascular function by activating the sirtuin-1 and nuclear factor erythroid 2–related factor-2 signalling pathways to increase nitric oxide production [11,12]. Additionally, the antioxidant effects of resveratrol may prove beneficial in hormonal migraine prophylaxis. Oxidative stress is a trigger of neurogenic inflammation, a key process in migraine pathophysiology, and antioxidants are thought to be useful in the prevention of migraine [8,13,14].

To our knowledge, no study has examined whether resveratrol supplementation can reduce the likelihood of hormonal migraine attacks occurring, improve migraine-related quality of life and/or reduce the migraine-related disability of hormonal migraineurs. Therefore, the aim of this study was to assess the effects of resveratrol supplementation on migraine frequency, migraine-related quality of life and migraine related disability.

## 2. Materials and Methods

### 2.1. Study Design

A 2 × 3-month, randomised, double-blind, placebo-controlled, crossover intervention trial that assessed the effects of daily resveratrol supplementation (75 mg twice daily) on the hormonal migraine burden index (HMBI), the migraine-related quality of life and the migraine-related disability of hormonal migraineurs was conducted.

Participants filled out a paper-based diary each day that collected information on their migraine attack characteristics and completed the following outcomes: the HMBI, the average hormonal migraine attack severity, the migraine-related disability questionnaires and the migraine-related quality of life questionnaire. 

All participants provided informed consent prior to enrolment in the study. The University of Newcastle Human Research Ethics committee approved the study protocol (H-2019-0416) and the clinical trial was registered with the Australian and New Zealand Clinical Trials Registry (ACTRN12620000180910).

### 2.2. Study Population

Participants were recruited nationwide using approved media advertising. Modes of advertising included radio, newspaper, social media platforms and the placement of flyers throughout the Hunter Region in NSW. Interested participants were provided with a detailed information sheet and completed a health and lifestyle questionnaire prior to enrolling in the study. 

Eligible participants were aged between 18 to 50 with a regular hormonal cycle length between 21 and 35 days and suffered from migraine, as defined by the International Classification of Headache Disorders-3 criteria [6], that occurred within three days from the onset of menstruation in their three previous menstrual cycles [15,16]. To be considered a migraine, participants were required to have a headache that lasted at least four hours with nausea or vomiting and at least two of the following characteristics: unilateral location, pulsating or throbbing pain, pain of at least moderate intensity (≥30 mm on a visual analogue scale), or pain aggravated by or causing avoidance of physical activity [6]. Interested volunteers were not eligible to participate in the trial if they had any of the following: amenorrhea, breastfeeding or pregnant, hysterectomy, fibromyalgia, polycystic ovarian syndrome, premature ovarian failure, insulin-dependent diabetes, liver or kidney disease, malignant cancer, neurological conditions (including stroke, TIA, multiple sclerosis, epilepsy, Chiari malformation or Parkinson’s disease), history of alcohol or drug abuse and unmanaged or untreated major depression. 

### 2.3. Intervention and Treatment Regimen

Resveratrol (Veri-te Resveratrol) and matching placebo capsules were provided by Evolva SA (Basel, Switzerland). Resveratrol capsules contained 75 mg of 98% trans-resveratrol and placebo capsules contained inactive excipients. Both resveratrol and placebo capsules were identical in terms of colour, shape, size, and packaging and were labelled with the participant’s study number. Treatment groups were randomised and allocated using Altman’s Allocation by Minimisation procedure [17] by an independent investigator who was not involved in the recruitment, screening or follow-up of participants. Treatment groups were balanced according to their age and average monthly frequency of hormonal migraine attack. Supplements were dispensed in sealed opaque containers of 200 capsules together with a supplement diary. Participants were instructed to consume one capsule of 75 mg resveratrol or matching placebo twice daily (morning and evening) with food for three months, after which they were allocated the alternate treatment to consume for a further three months. The dose of resveratrol was based on previous clinical research conducted by our research group where 75 mg of resveratrol supplementation twice daily was successful in improving cerebrovascular function [9,18]. A twice-daily dosing of resveratrol was deemed appropriate to maintain sufficient levels of blood plasma concentrations of resveratrol due to its short half-life following once-daily dosing (approximately 1–3 h) [19,20]. The total daily dose of resveratrol supplementation (150 mg/day) is lower than some previous studies [10]. However, only nanomolar concentrations of resveratrol are required for it to activate endothelial nitric oxide synthase [11]. Participants recorded their consumption of their allocated capsules daily in their study diary and the details of migraine attacks that they experienced during the treatment period, including the migraine attack duration, symptoms and pain intensity.

### 2.4. Study Outcomes

#### 2.4.1. Primary Outcome: Hormonal Migraine Burden Index 

The primary outcome for the study was the HMBI, which was calculated as the total number of days with hormonal migraine/number of hormonal cycles recorded during each 3-month phase of the study. The HMBI was extracted from a study diary which the participants were instructed to complete daily. A migraine was considered a hormonal migraine if it occurred ±3 days from the onset of menstruation, lasted for at least four hours, was accompanied by nausea and/or vomiting, or phonophobia and/or photophobia and had at least two of the following characteristics: unilateral location, pulsating and/or throbbing pain, pain aggravated by movement or measured at least 3 cm on the visual analogue scale for pain intensity [6,15,16]. 

#### 2.4.2. Secondary Outcomes

Visual Analogue Scale

Participants recorded the pain intensity of each hormonal migraine using a visual analogue scale with an 11-point scale (0–10). An average score was calculated as follows: total of individual visual analogue scores/number of hormonal migraines.

Migraine-Related Disability and Quality of Life Measures

Migraine-related disability was assessed using the Migraine Disability Assessment and the Headache Impact Test-6™ questionnaires. The Migraine-Specific Quality of Life version 2.1 was used to assess quality of life. The questionnaires were completed at three time points (baseline, month-3 and month-6). The questionnaires have been described in detail previously [7].

### 2.5. Sample Size

No previous study has looked at the effect of chronic resveratrol supplementation on hormonal migraine burden. The sample size calculation was, therefore, based on a desired medium-effect size difference of 0.5 between placebo and resveratrol for the hormonal migraine burden. In total, 65 completed data sets were needed to detect a significant (*p* < 0.05) difference in hormonal migraine burden between placebo and resveratrol at 80% power [14]. The mean attrition rate for a previous 6-month online migraine intervention study was 55% [21]. Therefore, we aimed to enrol a total of 145 women into this study.

### 2.6. Statistical Analyses

Analyses were performed using SPSS version 27.0 for Windows (IBM Corp., Armonk, NY, USA). The distribution of data was assessed using the Shapiro–Wilk test. Results are expressed as within-individual differences between outcome measures at the end of the 3-month resveratrol and placebo supplementation periods; significant differences were determined by paired *t*-test. For outcomes where the treatment difference was not normally distributed, a Wilcoxon signed ranks test was used. The treatment difference was the difference between the resveratrol and placebo treatment values. All data are presented as mean ± SEM unless otherwise noted.

## 3. Results

### 3.1. Baseline Characteristics

**Figure 1:**
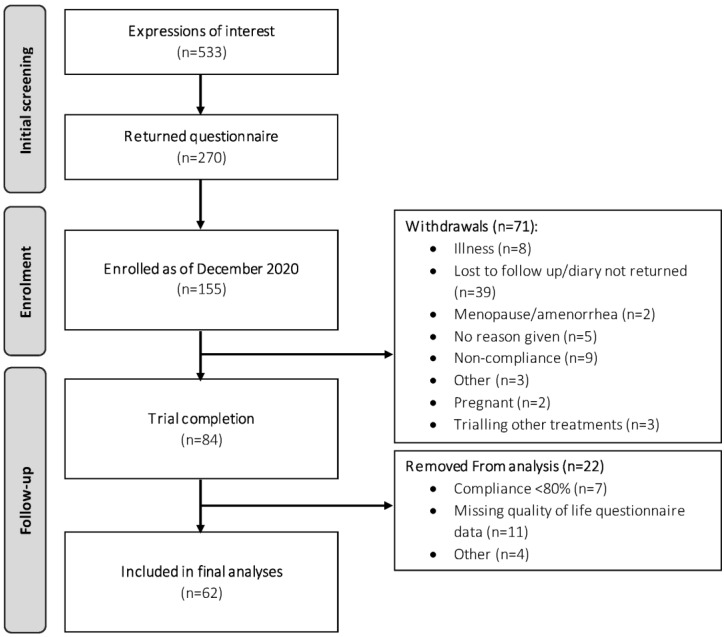
CONSORT diagram depicting flow of study participants.

The trial CONSORT diagram is shown in Figure 1. In total, 155 participants were enrolled into the study by the end of December 2020, of which 84 participants completed both phases of the trial. Of these 84 participants, 62 met the full study requirements (i.e., completion of questionnaires and compliance > 80%) and were included in the final analyses.

Baseline participant characteristics are shown in Table 1. At baseline, the average age of the participants included in the analyses was 37.5 ± 0.8 years, the estimated time since their first hormonal migraine was 11.4 ± 1.0 years and the estimated average days per month in which they experienced migraine was 3.1 ± 0.2 days. There were no statistically significant differences in participant demographics between the placebo and active groups at baseline (see Table 1). The baseline migraine-related quality of life and disability questionnaires indicated that the hormonal migraine cohort in this trial were experiencing severe migraine-related disability and that their hormonal migraines were having a moderate impact on their quality of life.

### 3.2. Compliance

Based on capsule count, overall compliance was 96% for both the placebo and resveratrol groups after the 3-month supplementation period. For crossover analysis, the average compliance was 95% and 97% for the placebo and resveratrol groups respectively.

### 3.3. Primary Outcome

There was no significant difference (*p* = 0.895) for the HMBI, the primary outcome, between the resveratrol and placebo treatments (Table 2). We also did not find any significant difference in the raw number of hormonal migraines between the placebo and resveratrol treatments.

### 3.4. Secondary Outcomes

#### 3.4.1. Visual Analogue Scale

There was no significant difference (*p* = 0.922) in the average hormonal migraine severity, measured by a visual analogue scale, between the placebo (3.92 ± 0.37) and resveratrol (3.94 ± 0.42) treatments.

#### 3.4.2. Migraine-Related Disability and Quality of Life

There was no significant difference in the migraine-specific quality of life domain score, the Headache Impact Test score or the Migraine Disability Assessment score between the placebo and resveratrol treatments (see Table 3).

#### 3.4.3. Responders and Non-Responders

Within-individual crossover comparisons revealed that after three months of resveratrol versus placebo supplementation, 29% of participants had a >50% reduction in their HMBI and 29% of participants had a >50% increase in the HMBI.

In terms of migraine-related disability, 52% of participants showed improvement (assessed by averaging the Headache impact test-6™ and Migraine disability assessment scores), 44% showed worsening, and 4% reported no change in their migraine-related disability.

Similarly, variables outcomes were found for quality of life, with 45% of participants experiencing improved quality of life, but 38% of participants experiencing a worsening and 17% of participants experiencing no change in migraine-related quality of life.

## 4. Discussion

In this randomised, double-blind, placebo-controlled, crossover trial, we found no statistically significant difference in the primary outcome, the HMBI, between the resveratrol and placebo treatments. Additionally, there was no difference between the resveratrol and placebo treatments for the secondary outcomes, which included average hormonal migraine severity, migraine-related quality of life, and migraine-related disability. Possible reasons for this lack of effect include inadequate trial duration, inappropriate dose of resveratrol or simply an inappropriate choice of resveratrol as a prophylactic agent for hormonal migraine. Furthermore, a recent review has reported that the health effects of resveratrol can be affected by a number of factors including an individual’s age, disease state, lifestyle and gut microbiome [22]. Therefore, it is possible that improvements of cerebrovascular function and neurovascular coupling that we have previously observed in older populations, including postmenopausal women [9] and in men and women with type 2 diabetes [18] may not be apparent in young, premenopausal women or hormonal migraineurs under the same treatment regimen.

There is limited research surrounding the potential role of phytoestrogen supplementation for the prevention of hormonal migraine. To date, only two studies have investigated whether phytoestrogen supplementation can reduce migraine frequency in hormonal migraineurs [23,24].

Ferrante et al. found that hormonal migraineurs taking an isoflavone mixture comprising 56 mg of genistein and 20 mg daidzein for ten consecutive days per month (from seven days prior until three days after the onset of menstruation) experienced a reduced frequency of migraine. However, their trial comprised only ten subjects and was not placebo controlled [23]; hence it is possible that other factors associated with participating in the trial may have resulted in their observed reduction over time as, in the present study, we observed a similar two-thirds reduction in migraine frequency from baseline to the end of each intervention phase, regardless of the type of treatment. The limitations of Ferrante et al.’s study highlight the strengths of our present study: a crossover trial to minimise variance which was placebo-controlled, randomised and double-blinded, to avoid allocation and selection bias. Furthermore, this study is the largest trial to date of phytoestrogen supplementation to treat hormonal migraine.

Burke et al. tested the effects of a mixture containing 60 mg of soy isoflavones, 50 mg of black cohosh and 100 mg of dong quai extract on migraine in a randomised, controlled trial in 38 hormonal migraineurs. They found that taking the phytoestrogen mixture twice daily for 24-weeks significantly reduced the frequency of hormonal migraines by 55% compared with placebo [24]. Whilst the trial design and the sample size in the study by Burke et al. are favourable, without a multi-arm study it is unclear as to which component(s) of the mixture (soy isoflavones, black cohosh or dong quai extract) contributed to this outcome. Whilst soy isoflavones are recognised as phytoestrogens [23], black cohosh and dong quai extract are not [23,25,26], yet they may have contributed to the reduction in hormonal migraine frequency with an unknown mechanism of action.

It is worth noting that the isoflavones used by Ferrante et al. and Burke et al., are ERβ-specific, whereas resveratrol, a stilbenoid polyphenolic compound, stimulates both oestrogen receptors, ERα and ERβ to increase nitric oxide bioavailability [11,12,23]. Interestingly, nitric oxide is a potent vasodilator that is implicated in the pathophysiology of migraine. When the trigeminovascular system is activated, nitric oxide, in combination with vasoactive neuropeptides, is released onto the cerebral and meningeal blood vessels. The release of nitric oxide and vasoactive neuropeptides causes vasodilation and consequent mechanical and chemical stimulation of sensory neurons of the trigeminovascular system, further perpetuating the migraine pain cycle [27,28]. Therefore, resveratrol supplementation could potentially trigger or worsen a migraine attack. However, our finding of no difference in the HMBI between placebo and resveratrol treatments in this present study does not support the latter. We hypothesised alternatively that resveratrol supplementation could improve endothelial vasomotor function in the cerebrovasculature, which we have previously found to be deficient in hormonal migraineurs [7], and thereby counteract migraine.

Despite the limitations of the studies by Ferrante et al. and Burke et al., and despite finding no difference in HMBI between placebo and resveratrol treatments in our present study, resveratrol and other phytoestrogen supplements may still have potential as prophylactic agents for hormonal migraine. Due to the current paucity of research surrounding phytoestrogen supplementation in hormonal migraine, further longitudinal intervention studies that assess changes in cerebrovascular function are needed to confirm or rule out phytoestrogen supplementation as a potential prophylaxis for hormonal migraine.

In this present study, our baseline migraine-related quality of life and migraine-related disability scores were, for the most part, comparable to our previous cross-sectional study that was conducted in 50 hormonal migraineurs [7]. Our findings from the present study indicate that hormonal migraine was having a severe impact on quality of life and a moderate impact on disability in our cohort, further highlighting the detrimental impact of hormonal migraine. Our findings of no difference in migraine-related quality of life and disability between the resveratrol and placebo treatments and of similar percentages for responders and non-responders to resveratrol align with our finding of no difference in the HMBI between resveratrol and placebo treatments. It is important to note that the questionnaires used in this study may have been subject to recall bias, particularly in the case of the Migraine Disability Assessment questionnaire as it assesses migraine-related disability over a period of three months. Therefore, analyses involving our migraine-related disability and quality of life questionnaires may be limited.

## 5. Conclusions and Future Directions

To conclude, this is the first study to investigate the effects of a 3-month supplementation of resveratrol (150 mg/day) on HMBI. We found that this treatment did not improve or worsen HMBI, average hormonal migraine severity, migraine-related quality of life or migraine-related disability in our cohort of hormonal migraineurs. Whilst we were not able to find an improvement in the HMBI or quality of life and disability measures following this dose and duration of resveratrol supplementation, our findings do not rule out the potential for resveratrol at a different dose or different supplementation period to alter these outcomes in hormonal migraine. Further studies are also needed to confirm the effect of resveratrol supplementation on the cerebrovascular function of hormonal migraineurs, as this remains an unanswered question.

## Figures and Tables

**Table 1:** Participant Characteristics.

Characteristics	All Participants (*n* = 62)	Participants Initially Allocated to		
		Placebo (*n* = 31)	Resveratrol (*n* = 31)	*p* Value
Age (years)	37.5 ± 0.8	37.4 ± 1.1	37.6 ± 1.1	0.887
Years with hormonal migraine ^†^	11.4 ± 1.0	11.5 ± 1	11.3 ± 14	0.915
Menstrual cycle length ^†^	28.4 ± 0.2	28.4 + 0.4	28.3 + 0.3	0.877
Headache impact test-6™	63.5 ± 0.7	63.9 ± 1.2	63.1 ± 0.9	0.395
Migraine disability assessment				
Total score	19.6 ± 1.8	18 ± 1.9	21.1 ± 3.2	0.433
Item A	11.8 ± 0.9	11.2 ± 1.2	12.4 ± 1.4	0.575
Item B	6.6 ± 0.2	6.5 ± 0.3	6.7 ± 0.3	0.511
Migraine-specific quality of life				
Emotional function domain	48.8 ± 3.5	48.4 ± 5.4	49.2 ± 4.6	0.914
Role function preventive domain	60.3 ± 3.1	61.9 ± 4.2	58.7 ± 4.7	0.626
Role function restrictive domain	44.1 ± 3.0	46.3 ± 4.5	41.9 ± 4.0	0.496
Estimated HMBI (migraine days/month)	3.1 ± 0.2	3.0 ± 0.2	3.1 ± 0.3	0.862
Contraception use (yes)	16 (26%)	8 (26%)	8 (26%)	1.000
Smoking history (yes)	10 (16%)	4 (13%)	6 (19%)	0.490
Guessed allocation correctly (%) ^‡^	35 (56%)	15 (48%)	20 (65%)	0.205

^†^ Based on recall. ^‡^ Independent *t*-test. The Headache impact test-6™ score ranges between 36–78; a higher the score represents a greater impact of migraine on an individual’s ability to function at work, home and social settings. The total Migraine disability assessment score ranges between 0–270; a higher the score represents a greater migraine-related disability. Migraine disability assessment item A and item B represent the average migraine frequency and the average pain intensity during the previous three-month period, respectively. A higher Migraine-specific quality of life domain score represents a better quality of life (self-perceived).

**Table 2:** Migraine Characteristics.

Characteristics	Crossover Comparison			
	Placebo (*n* = 62)	Resveratrol (*n* = 62)	Treatment Difference (Resveratrol–Placebo)	*p*-Value
Raw number of hormonal migraines per phase	1.03 ± 0.13	1.07 ± 0.14	0.032 ± 0.177	0.917
Hormonal migraine burden index	0.56 ± 0.09	0.56 ± 0.09	−0.003 ± 0.099	0.895

Wilcoxon Signed-Rank Test used for all comparisons.

**Table 3:** Migraine-Related Disability and Migraine-Specific Quality of Life.

Characteristics	Crossover Comparison			
	Placebo (*n* = 62)	Resveratrol (*n* = 62)	Treatment Difference (Resveratrol–Placebo)	*p*-Value
Headache impact test-6™	59.4 ± 0.9	58.7 ± 1.1	−0.7 ± 0.9	0.491
Migraine disability assessment				
Total score	15.6 ± 2.3	12.6 ± 1.8	−3.0 ± 2.4	0.367
Item A	9.9 ± 1.0	8.7 ± 0.8	−1.3 ± 0.9	0.341
Item B	5.2 ± 0.2	5.4 ± 0.2	0.2 ± 0.3	0.587
Migraine-specific quality of life				
Emotional function	62.9 ± 3.8	67.1 ± 3.6	4.2 ± 4.2	0.253
Role function preventive	69.9 ± 3.5	75.3 ± 3.3	5.4 ± 3.3	0.106
Role function restrictive	57.8 ± 3.6	62.7 ± 3.6	4.9 ± 3.6	0.239

Wilcoxon Signed-Rank Test used for all comparisons except the Headache Impact Test.

## Data Availability

All data for this study are contained within the article.
